# Environmental Changes to Promote Physical Activity and Healthy Dietary Behavior

**DOI:** 10.1155/2012/470858

**Published:** 2012-09-06

**Authors:** S. P. J. Kremers, F. F. Eves, R. E. Andersen

**Affiliations:** ^1^Department of Health Promotion, NUTRIM School for Nutrition, Toxicology and Metabolism, Maastricht University Medical Centre, 6200 MD Maastricht, The Netherlands; ^2^School of Sport & Exercise Sciences, the University of Birmingham, Birmingham B15 2TT, UK; ^3^Department of Kinesiology and Physical Education and School of Medicine, McGill University, Montreal, QC, Canada H2W 1S4

This special issue is devoted to the study of environments that make physical activity and/or healthy dietary behaviors more likely. Empirical evidence regarding the influence of environmental factors on physical activity and dietary behavior is growing rapidly. The evidence base, however, is typically built on (cross-sectional) studies, based on self-reports. There is a dearth of studies with a longitudinal design and proper measurements of the environment, as well as studies that apply isolated (small-scale) environmental manipulations or those that involve (large-scale) intersectoral collaborations in the implementation of sustained environmental interventions. The papers in this special issue address a wide variety of studies towards environmental influences on dietary behavior and physical activity. For example, studies in this special issue are aimed at developing good measurement instruments, applying systematic observations, longitudinal research designs, or focusing at environmental interventions and intersectoral collaboration. Multiple distinct target groups, settings, and behaviors are examined. The effects on children and adolescents as well as adults were included in this special issue. Some papers concern only the physical activity environment or only the dietary behavior environment, while other investigations reflect environments with a focus on both physical activity and diet. While reading the special issue, the reader will note that the settings investigated include the neighborhood environment, the school environment, child care, health care setting, food stores, and local government.

Studies such as those presented in this volume will help us design interventions and health policies that change the environment in order to make physical activity and healthy dietary behavior more likely. In this respect, it is encouraging to realize that relatively small changes to cues in the physical environment may induce relatively large behavioral changes. Environmental changes, sometimes referred to as “nudges”, can be separated meaningfully into two different approaches. Passive nudges involve changes to the choice architecture of the environment to bias choices away from unhealthy options. Thus, changing the layout of cafeteria food [1] or even the positioning of items on the menu [2] can bias behavior towards more healthy choices. Similarly, stairs that are reached before an escalator are more likely to be chosen by pedestrians leaving a station on the way to work [3]. Of these two examples, it is clear that retrofitting the physical activity landscape towards more physically active choices would entail considerably more costs than reconfiguring the choice architecture of the canteen environment. Conversely, changing the pricing structure of meal choices (e.g., [4]) entails greater costs than changing the speed at which elevators transport individuals within a building [5] or restricting the number of floors at which elevators stop [6] to promote stair usage. Environmental changes cannot be considered in isolation from issues of costeffectiveness, and the latter often requires action at a policy level. 

Active environmental nudges involve positioning of prompts in the environment at the time choice is made to encourage more healthy choices. Thus, point-of-purchase labeling of calorific content of food (e.g., [7]) and point-of-choice prompts for stair climbing (e.g., [8]) provide health relevant information in the environment when the choice is considered. We term these “active” nudges because they remind individuals of the health plans that they may have made before encountering the choice point. As such, active nudges require individuals to have a prior intention or plan to change behavior [8, 9] and the prompts remind individuals of these prior plans at the time choice is made. These active nudges can link to health promotion policy at the government level that disseminates information on behavior and health. Changes to the visibility of healthy alternatives such as prominent displays of fruit and vegetables can also be termed active in that they can link to prior planning, though they provide no explicit reminder about an individual's prior planning to encourage active consideration of the healthy alternative to counter the prominent effects of taste on food choice [10]. In contrast, curtailing the provision of sweets at the supermarket checkout removes cues to consumption in the environment, in effect, passively nudging consumers away from unhealthy choice by removing it. Of course, curtailing availability of cues or enforcing menu labeling (e.g., [7]) requires action at the policy level.

Environmental changes in microenvironmental settings (e.g., supermarkets, schools, worksites, neighborhoods, and restaurants) can be divided in different types [11]: physical, social-cultural, economic, and political environmental changes, often with interlinkage between the different spheres of influence [12]. An overview with examples is provided in Table 1. Although the table is not systematically constructed and cannot be considered as complete, it does show that environmental changes consist of a wide range of possibilities. Some of the environmental changes mentioned in the table can be considered as “drastic” (e.g., punishing policies for unhealthy choices such as car use [13]), other examples can be considered as “subtle” (e.g., footsteps as prompts to promote stair use [14]). Most of the mentioned changes have been empirically studied in one or more relevant areas. These studies generally show small to moderate effect sizes, especially when they are used in combination with behavioral-didactic intervention components that might be expected to promote planning for a healthier lifestyle. Moreover, often relatively small environmental changes in specific settings, such as increasing the availability of healthy choices in worksites, can be regarded as an effective first step towards sustained interventions (e.g., by integrating them into policies). Note that relatively few studies have formally assessed changes to the economic environment (e.g., [4]) as well as studies assessing the impact of changes in policy (e.g., to prepare and offer healthy foods). Policies need to be written, approved, implemented, promoted, and sustained. Moreover, they are more successful when they have been formed on the basis of an integrated collaborative process among decision makers. Evaluation of a policy intervention should follow this entire process, making such interventions more difficult to evaluate than other types of environmental interventions, which may account for the lack of such studies in the literature.

Observational research towards the influence of environmental influences has often applied an isolated approach, while an ecological perspective would be more appropriate to understand the complex dynamics underlying physical activity and dietary behavior. The ecological perspective of health behavior has been central to public health concepts and methods since the nineteenth century. In a broad sense, the term “ecology” refers to the interrelations between organisms and the environments they inhabit. One feature of ecological models needs extra attention here: context. The feature of context refers to multiple spheres of the social, physical, economic, and political environment (micro-, meso-, exo-, and macrosystems) that influence behavior. Ecological studies in the field of child development have shown that the impact of microlevel factors (e.g., parental support for a child to play outside) on individual behavioral developmental variability can vary as a function of contextual macrolevel conditions (e.g., the presence of playgrounds in the neighborhood). The existence of such “higher order moderation” has also been suggested in the field of physical activity and dietary behavior [15–19]. The operation of higher order moderation processes underlines the importance of distal, so-called “upstream” determinants of physical activity and dietary behavior, that, to date, have mostly been operationalized as confounders in causal chain determinants research. In contrast, a contextual rather than mechanistic orientation in operationalizing such broader environments will bring us further in explaining and predicting changes in physical activity and dietary behavior. 

*S. P. J. Kremers* *S. P. J. Kremers* *F. F. Eves* *F. F. Eves* *R. E. Andersen* *R. E. Andersen*

## Figures and Tables

**Table 1:** Physical, social-cultural, economic, and political environmental changes to promote healthy dietary behavior and physical activity (PA).

	Physical	Social-cultural	Economic	Political
Food	Nutrition labeling Point-of-purchase prompts Placing of healthy foods in more prominent places Increasing availability and accessibility of healthy foods Decreasing the availability and accessibility of unhealthy foods Restricting the use of logos Restricting the scheduling of commercials promoting unhealthy food to children	Providing social norm feedback Increasing visibility of healthy role models Facilitating healthy food group activities	Decreasing the price of healthy food Increasing the price of unhealthy food	Implementing policies towards the provision of physical, social, and economic environmental changes Rewarding healthy food choices Punishing unhealthy food choices Changes in the laws governing advertising

Physical activity	Point-of choice prompts Tailoring prompts to target certain populations Increasing attractiveness of the PA environment Decreasing the availability and accessibility of inactive choices Placing of active alternatives (e.g., stairs) at more prominent places Increasing visibility of active alternatives Increasing availability and accessibility of active alternatives	Providing social norm feedback Increasing visibility of healthy models Facilitating group PA activities	Decreasing price of structured PA activities Increasing the cost of car parking Congestion charging in major cities Fair subsidies for public transport	Implementing policies towards the provision of physical, social, and economic environmental changes Provision of pedestrianized areas Rewarding physically active alternatives Punishing choices for physically inactive alternatives Transport planning
